# P036. Headache attributed to non-traumatic intracranial bleeding

**DOI:** 10.1186/1129-2377-16-S1-A174

**Published:** 2015-09-28

**Authors:** Rita Lucia Trinchi, Francesco Migliorini, Francesco Odoguardi

**Affiliations:** Centro Cefalee, Ospedale G. Chidichimo, Trebisacce, CS Italy; U.O. Radiologia, Ospedale G. Chidichimo, Trebisacce, CS Italy

## Classification

Headache attributed to non-traumatic intracranial bleeding is classified by the ICHD-2 and the ICHD-3 Beta criteria at code 6.2.1.

In the ICHD-2 classification at code 6.3.4 is classified headache attributed to cavernous angioma as a result of intracerebral bleeding, while in the ICHD-3 beta version at the same code, the headache is closely linked to vascular malformation and not bleeding [1, 2].

## Case report

A male patient, 37 years old, married with 2 children, came to our observation with a history of 7 days of headache.

Seven days before, at 3.30 am, he experienced a sudden onset of headache which in 2-3 minutes became severe, accompanied by paresthesia of the left upper limb, chest, right upper limb, lower limb, with tightness of the throat, unconsciousness and spreading of tremors.

The patient was taken with an ambulance to the nearest Spoke Center, where a Stress Syndrome was diagnosed and treated with BDZ and discharged at 12.00 am.

In the following three days the patient reported a state of drowsiness and headache lasting for 6-7 hours daily, bilateral, at the temporal level, of pulsating nature, accompanied by sweating, photophobia, and phonophobia. Physical effort was a trigger factor.

Given the brief medical history and absence of diseases worthy of note, the patient underwent imaging techniques: first CT and CT angiography, which excluded pathologies, then RMN and AngioRMN with and without contrast medium which showed a cavernous angioma in the left juxtacortical occipital, with signs of intralesional bleeding.

## Conclusions

The patient bearer of cavernous angioma had never previously suffered from headaches, and current symptoms are closely related to intralesional bleeding.

Written informed consent to publish was obtained from the patient(s).
